# Attentional Bias and Training in Individuals With High Dental Anxiety

**DOI:** 10.3389/fpsyg.2020.01057

**Published:** 2020-06-09

**Authors:** Jedidiah Siev, Evelyn Behar, Meghan R. Fortune

**Affiliations:** ^1^Department of Psychology, Swarthmore College, Swarthmore, PA, United States; ^2^Department of Psychology, Hunter College, The City University of New York, New York, NY, United States; ^3^Institute for Health Research and Policy, University of Illinois at Chicago, Chicago, IL, United States

**Keywords:** dental anxiety, attentional bias, attention training, information processing, imagery

## Abstract

Dental anxiety is common and associated with negative outcomes. According to information-processing models, anxiety is maintained by maladaptive patterns of processing threatening information. Furthermore, attention training interventions can reduce anxiety in one session. Fifty-three individuals with high levels of dental anxiety completed a Posner reaction-time task. Participants were randomized to attention training or control using a dot-probe task, and then attentional bias was remeasured using another Posner task. Participants then completed a script-driven imaginal exposure task. Results indicated that individuals high in dental anxiety exhibit threat-relevant attentional bias. There was mixed evidence about the efficacy of attention training. On the one hand, training did not eliminate attentional bias and training condition did not predict distress during the imagery task. On the other hand, cue dependency scores in the control group were higher for dental than neutral cues, but did not differ in the training group. In addition, cue dependency scores for both dental and neutral cues predicted subjective anxiety in anticipation of the imagery task. The mixed results of training are considered in terms of the possibility that it enhanced attentional control, rather than reducing bias.

## Introduction

Dental anxiety is experienced by approximately 10–20% of adults, with 2.4–3.7% of the population meeting diagnostic criteria for dental phobia (Stinson et al., 2007; Oosterink et al., 2009). Anxiety about dental procedures is a predictor of avoidance of seeking dental care (e.g., Pavi et al., 1995), with more than two-thirds of individuals with high dental anxiety reporting that they avoid or delay dental visits (e.g., Armfield and Ketting, 2015). Such avoidance can ultimately lead to the onset or exacerbation of poor oral health (e.g., Doerr et al., 1998). According to the Centers for Disease Control and Prevention, 23% of adults between the ages of 20 and 64 have untreated dental caries (National Center for Health Statistics, 2011), and dental fears are the reason an estimated 23 million adults in the United States avoid dental care (Dionne et al., 1998). Therefore, not surprisingly, dental anxiety is associated with higher rates of dental caries and dental neglect (e.g., Poulton et al., 1998), as well as higher rates of periodontal disorders (Luca et al., 2014). Indeed, 75% of dentists identify anxiety as an important cause of inadequate dental care in the population (O’Shea et al., 1984). Moreover, poor oral health is associated with other health outcomes including systemic diseases, stroke, heart disease, poor nutrition, and impaired social activities (U. S. Department of Health and Human Services, 2000; Dye et al., 2007).

Considering the high prevalence of dental anxiety and the associated public health cost, there is a paucity of experimental research on the psychological mechanisms associated with dental anxiety and its treatment. This is especially striking when considering that anxiety in general is among the best understood and most effectively treated domains of psychopathology. With regard to research on the mechanisms that might cause or maintain dental anxiety, the majority of investigations have relied on self-report questionnaires to examine cross-sectional relationships between dental anxiety and variables of interest, with markedly fewer studies employing laboratory and/or experimental methods.

According to information processing theories, anxiety is maintained by maladaptive patterns of processing threatening information, such as selective attention toward threat and difficulty disengaging from threat (e.g., Mogg et al., 1991). That is, anxious individuals pay too much attention to the things they fear, and they experience difficulty redirecting their attention away from those things. Basic research in the broader anxiety literature has demonstrated that individuals with anxiety disorders or sub-syndromal anxiety symptoms evidence elevated vigilance toward threatening information (for reviews, see Bar-Haim et al., 2007; Mogg and Bradley, 2016). One early study further demonstrated that training individuals to attend to threatening stimuli leads to enhanced emotional reactivity during a subsequent stressful task, providing crucial evidence that attentional bias toward threat is causally related to emotional disturbance (MacLeod et al., 2002).

Studies documenting attentional bias toward threat among anxious individuals have led to the development of cognitive bias modification interventions that train attention away from threat with the goal of reducing emotional distress. A series of studies demonstrate the efficacy of these approaches in reducing attentional threat biases in a wide array of conditions, including generalized anxiety disorder (Amir et al., 2009a), social anxiety disorder (e.g., Amir et al., 2008; Schmidt et al., 2009; Amir et al., 2009b), and subclinical obsessive-compulsive disorder (OCD; Najmi and Amir, 2010). Extant studies examining attentional bias retraining procedures suffer from two important limitations. First, the majority of these investigations have utilized the same procedure for *assessing* attention as they did for *training* attention, which might mean that participants simply became more skilled at the task (Amir et al., 2009b). Second, although many investigations have examined the efficacy of these attention retraining paradigms for reducing attentional threat, fewer have investigated whether these reductions in attentional bias generalize to outcomes other than cognitive bias itself. Najmi and Amir (2010) found that after only one session of attention training, anxious individuals were more willing to approach feared stimuli even when they did not experience reduced anxiety while doing so, but the majority of studies do not include behavioral approach tasks in order to examine generalizability of findings to non-cognitive outcomes.

In the dental anxiety literature, several investigations have likewise sought to examine the role of attentional bias in dental fears. Three of these investigations have utilized the Stroop procedure to examine whether individuals with dental anxiety exhibit attentional bias toward threat. In two studies of individuals with high dental anxiety, participants were slower to name words depicting dental anxiety-related threat relative to those with low dental anxiety (Muris et al., 1995), and compared to neutral words (Johnsen et al., 2003). In another study of individuals with diagnosed dental phobia, Ries (1997) found that relative to demographically matched non-anxious participants, participants with a dental phobia diagnosis were slower to name both standardized and idiographic words depicting dental threat. Such attentional biases indicate an interference of the attentional system such that cognitive resources are allocated to feared stimuli^1^. Notably, because the emotional Stroop paradigm requires participants to state the color of a word when that word itself depicts dental fears, it is possible that increased negative affect (NA; as opposed to increased attention) is responsible for longer reaction times (MacLeod et al., 1986). In contrast, other tests of attention allocation (e.g., probe detection paradigms) require participants to detect non-emotional stimuli that replace either threat-related or neutral stimuli, and thus reduce the risk of reaction times varying with emotional state. To complicate things further, there is recent evidence that individuals with dental phobia and a history of traumatic experiences associated with dental treatment evince patterns of neural activation that differ from individuals with other anxiety disorders when exposed to threat-relevant pictures (Alexopoulos et al., 2019).

Importantly, no studies to date have examined the potential efficacy of attention training for dental anxiety among individuals high in dental anxiety (cf. Horovitz et al., 2016, who excluded such individuals). This gap in the scientific literature is underscored by the possibility that a brief computerized intervention could be especially helpful for people with high dental anxiety to increase their willingness to complete dental procedures they would otherwise avoid. Although a few studies have demonstrated that brief cognitive and behavioral interventions can be helpful in reducing dental fear (e.g., Jerremalm et al., 1986; Getka and Glass, 1992; de Jongh et al., 1995a; Haukebø et al., 2008; Gordon et al., 2013), and that such interventions are associated with lower relapse rates relative to benzodiazepine treatment (Thom et al., 2000), dentists are generally unable to provide these brief interventions (e.g., Gujjar et al., 2019). Dentists are unlikely to have the training, resources, and interest in implementing cognitive and behavioral interventions, and thus instead frequently offer patients sedation (e.g., benzodiazepine, nitrous oxide; Woodmansey, 2005). Dentists who do want their patients to utilize psychosocial interventions must appeal to liaison psychiatry and psychology services to provide such interventions (Feinmann and Harrison, 1997; Woodmansey, 2005). Even so, successful implementation of cognitive and behavioral interventions is predicated on numerous conditions, including patient willingness to seek adjunctive treatment, patient motivation to treat symptoms to remission (as opposed to temporarily managing distress sufficiently to complete a dental procedure), availability of resources, and cost of care being non-prohibitive or deterring. Therefore, there is a great need for portable, cost-effective, brief interventions to help patients with dental anxiety manage their anxiety in anticipation of a procedure, particularly interventions that could realistically be implemented in dental clinics without involving other specialists. Such an intervention could ultimately lead to a notable public health benefit. Indeed, there is some, albeit limited, evidence that brief computerized (e.g., Heaton et al., 2013) or other technology-based interventions can be helpful in reducing dental anxiety (e.g., Gujjar et al., 2019).

In this study, we examined two primary questions. First, we sought to assess whether individuals with dental anxiety evidence an attentional bias toward dental anxiety-relevant stimuli. Second, we examined the efficacy of a single-session attentional bias training procedure for reducing both attentional bias and self-reported distress during a behavioral (imaginal exposure) task, using different paradigms to measure and train attention.

## Materials and Methods

### Participants

Participants were recruited through various methods and sources, including (1) advertisements posted at the Departments of Psychology and Schools of Dentistry at Nova Southeastern University and the University of Illinois at Chicago (UIC); (2) advertisements sent to private dental clinics in South Florida and Chicago; and (3) e-mails sent to all university students, faculty, and staff at the UIC. Advertisements specified the need for individuals who were afraid of going to the dentist, and instructed participants to contact study personnel for a telephone screen. Telephone screening procedures entailed verbal administration of the Modified Dental Anxiety Scale (MDAS; Humphris et al., 1995); screening questions regarding psychiatric symptoms, substance use, and suicidality; questions regarding current or past psychological or psychiatric treatment; and demographic items.

A total of 58 individuals with MDAS scores of 19 or higher were invited to participate and consented. Of these individuals, 53 participants completed the study and were compensated $50. One participant’s MDAS score had dropped to 18 at the time of her participation but was nevertheless included. Participants were excluded if they had a psychotic disorder, bipolar disorder, current substance abuse or dependence, homicidality, or suicidality. They were also excluded if there was evidence of intellectual disability, dementia, brain damage, or other cognitive impairment. Four participants who consented were found ineligible due to current substance dependence, and another one withdrew. Table 1 presents demographic and symptom information for all participants^2^. Independent samples *t*-tests indicated that the experimental groups did not differ in self-reported symptoms of depression (*p* = 0.11, *d* = 0.45), anxiety (*p* = 0.35, *d* = 0.26), or stress (*p* = 0.52, *d* = 0.18).

**TABLE 1:** Demographic and clinical composition by group.

	**Attention training (*n* = 27)**	**Control (*n* = 26)**	**Entire sample (*N* = 53)**
Age *M* (SD)	39.41 (15.09)	40.54 (14.96)	39.96 (14.89)
Gender *n* (%) women	23 (85.2%)	19 (73.1%)	42 (79.2%)
Race			
Caucasian *n* (%)	11 (40.7%)	20 (76.9%)	31 (58.5%)
Black or African-American *n* (%)	9 (33.3%)	2 (7.7%)	11 (20.8%)
Asian or Asian-American *n* (%)	6 (22.2%)	4 (15.4%)	10 (18.9%)
Other *n* (%)	1 (3.7%)	0 (0%)	1 (1.9%)
Ethnicity *n* (%) Hispanic/Latino	4 (14.8%)	6 (23.1%)	10 (18.9%)
MDAS *M* (SD)	22.15 (1.49)	21.73 (1.61)	21.94 (1.55)
ADIS Interference *M* (SD)	5.04 (1.63)	4.83 (2.16)	4.94 (1.88)
ADIS Distress *M* (SD)	5.48 (1.31)	5.46 (1.48)	5.47 (1.38)
DASS-21 Depression *M* (SD)	5.48 (6.51)	8.62 (7.37)	7.02 (7.06)
DASS-21 Anxiety *M* (SD)	7.07 (6.39)	9.01 (8.55)	8.03 (7.52)
DASS-21 Stress *M* (SD)	12.67 (11.23)	14.46 (8.70)	13.55 (10.01)

*MDAS, Modified Dental Anxiety Scale; ADIS, Anxiety Disorders Interview Schedule; and DASS-21, Depression Anxiety Stress Scales-21.*

### Diagnostic Interviews

#### Mini International Neuropsychiatric Interview—Version 5.0 (MINI; Sheehan et al., 1998)

The MINI is a structured diagnostic interview based on the diagnostic criteria in the *DSM-IV*. It was developed to meet the need for a short but accurate structured interview for clinical and epidemiological studies, and assesses the presence of depression and dysthymia, manic and hypomanic episodes, anxiety disorders, alcohol and substance abuse/dependence, psychotic disorders, anorexia, bulimia, and antisocial personality disorder. The MINI also allows for diagnostic assessments made by interviewers with limited training. When comparing diagnoses using the MINI to those with the Structured Clinical Interview for DSM-III-R (SCID; Spitzer et al., 1990), the degree of concordance evidences a range according to the method of comparison and disorder, with kappa agreements ranging from 0.43 to 0.90, sensitivity values ranging from 0.45 to 0.96, and specificity values ranging from 0.86 to 1.00 (Sheehan et al., 1998).

#### Anxiety Disorders Interview Schedule—Specific Phobia Module (ADIS; Brown et al., 1994)

The ADIS is a semistructured interview for the assessment of anxiety disorders, depressive disorders, and substance disorders. Due to the MINI’s relatively weak concordance with SCID diagnoses of specific phobia, we also sought to assess dental phobia using a more comprehensive interview, the ADIS. Due to an administrative error because of which requisite clinical severity ratings were not assigned at one site, formal diagnoses were not recorded; however, interviewers assigned 0 (none) to 8 (very severe) ratings of both interference and distress related to dental fear; 96% of the participants received an interference or distress score of at least 4, indicating that they likely met the criteria for current dental phobia.

### Self-Report Measures

#### Modified Dental Anxiety Scale (MDAS; Humphris et al., 1995)

The MDAS is a five-item measure of dental anxiety designed to assess anticipatory anxiety associated with an upcoming dental visit, fear of dental cleaning and drilling, and receiving a local anesthetic injection. Total scores range from 5 to 25. The MDAS demonstrates good internal consistency (Humphris et al., 2000) and retest reliability (Humphris et al., 1995). It also demonstrates good convergent validity with scales of dental avoidance (Humphris et al., 2000). A cutoff score of 19 maximizes sensitivity and specificity in detecting cases and non-cases of dental phobia (Humphris et al., 1995; King and Humphris, 2010). We do not report α in the present sample because all participants had a minimum score of 19 on the MDAS, leading to a severely restricted range (19–24). This has been demonstrated to produce biased and sometimes even negative α values (Fife et al., 2012).

#### Depression Anxiety Stress Scales (DASS-21; Lovibond and Lovibond, 1995)

The DASS-21 consists of three seven-item scales that measure symptoms of depression, anxiety, and stress. Evidence indicates that the DASS-21 distinguishes well between these three features (Antony et al., 1998). It evidences good internal consistency (Lovibond and Lovibond, 1995), as well as concurrent validity (Antony et al., 1998). The DASS-21 was included to ensure that levels of depression, anxiety, and stress did not differ between the two experimental conditions. Internal consistency in the current sample was acceptable (depression α = 0.84; anxiety α = 0.75; and stress α = 0.88).

#### Positive and Negative Affect Schedule (PANAS; Watson et al., 1988)

The PANAS is a 20-item measure that assesses positive affect (PA) and NA. The Moment version of the PANAS involves rating affect in the present moment on a 1 (very slightly or not at all) to 5 (extremely) scale. The PANAS-Moment has good internal consistency and favorable convergent, discriminant, and predictive validities (Watson et al., 1988). Rather than creating scores for analysis, the PANAS was used to identify emotions for use in the imagery scripts.

### Experimental Tasks

#### Stimuli

A total of 24 dental images and 24 neutral images were selected for use in the attention tasks, so that a different set of 8 dental and 8 neutral images was used in each of the three tasks. Dental images were pictures of dental procedures and apparatuses, in most cases including tools in use by a dental professional on a patient. Several were chosen from the International Affective Picture System (IAPS; Lang et al., 2008; pictures 2279, 3280, and 9582) and others from Google Images. Neutral images depicted individuals working with tools or other similar technical implements, chosen to mirror the dental images; they were also selected from Google Images. The final set of images was selected from a larger pool of images following pilot testing using the valence and arousal dimensions of the Self-Assessment Manikin (SAM; Bradley and Lang, 1994), which are scored 1 to 9, with a middle score of 5 indicating neutrality. Neutral images were selected to be the most neutral in terms of valence. In contrast, dental images were selected to be the most extreme in order to (a) generate more variance in participants’ responses to them and (b) test whether attention training away from threat would be effective even when the threat image is more negative or more arousing than the neutral image. Mean valence scores from the pilot data were 3.67 (SD = 0.62) for dental pictures and 5.09 (SD = 0.38) for neutral pictures, indicating that the neutral pictures were rated as almost exactly neutral. Mean arousal scores from the pilot data were 5.73 (SD = 1.05) for dental pictures and 4.18 (SD = 1.30) for neutral pictures. Images are available from the first author.

#### Assessment of Attentional Bias

To assess attentional bias toward dental stimuli, we used the Posner paradigm (Posner, 1980; Fox et al., 2001; Yiend and Mathews, 2001), which has been used to measure attentional bias in previous studies of anxiety disorders (e.g., Amir et al., 2003). Participants were instructed to fixate on a cross at a central point on a computer screen (1,000 ms), and then a threatening or neutral image appeared (600 ms) in a rectangular box to the left or right of the fixation point. In order to avoid masking the target probe, the picture then disappeared for 50 ms, after which a probe (an asterisk) appeared in one of the two boxes. Participants indicated “as quickly as possible” where the asterisk appeared. The probe remained visible for 3,000 ms for trials on which a participant did not respond, and the intertrial interval varied from 500 to 1,500 ms. In valid trials (2/3 of the trials), the probe appeared in the location of the cue; in invalid trials (1/6), it appeared on the other side of the cue; in other trials (1/6), there was no cue. Relative facilitation to detect probes that follow in the location of threatening images, as well as relative delays to detect probes that appear in the position opposite threatening images, indicate attentional bias.

After eight practice trials, participants completed 192 trials in each of two Posner task administrations (pre- and post-experimental manipulation). In each administration, different sets of eight dental pictures and eight neutral pictures were presented 10 times each, and 32 trials were uncued (in which case the interval between the onset of the trial and presentation of the probe was 1,600 ms). Although not included in the analyses, uncued trials are typically presented to avoid a fixed cue interval (e.g., Amir et al., 2003).

#### Attentional Bias Modification

Attentional bias training was modeled after the probe detection paradigm (MacLeod et al., 1986), as well as after prior investigations that sought to train attention away from anxiety-relevant cues (e.g., Najmi and Amir, 2010). In this probe detection paradigm, participants viewed a fixation cross for 500 ms. Two pictures then appeared for 500 ms, one above the other, after which a probe (the letter “E” or “F”) appeared in the location of one of the pictures. Participants were instructed to indicate “as quickly as possible” whether the letter was an “E” or an “F.” Shorter response latencies imply that a participant’s attention was already engaged at the location of the probe. Therefore, decreased latency to identify the probe when it followed the location of threat, relative to neutral images, is evidence for attentional bias toward threat. However, in the present study, attentional bias was assessed using the Posner paradigm, and this task was used only as an experimental manipulation.

In the *attention training* condition, the location of the probe was contingent upon the location of the threat such that it always followed the location of a neutral image, thereby training participants to direct their attention away from threat in threat–neutral pairs. In the *control* condition, the location of the probe appeared equally in the previous location of the threat and neutral images. Researchers have demonstrated the remedial effects of attention training using the probe detection paradigm as the training mechanism (e.g., Schmidt et al., 2009; Amir et al., 2009a; Najmi and Amir, 2010).

After 10 practice trials with neutral pictures, participants completed 192 trials in which a dental picture was always paired with a neutral picture. There were eight dental pictures and eight neutral pictures, and each picture appeared 24 times in total. The pictures were different from those used in the Posner tasks, and the intertrial interval was 1,000 ms.

#### Construction of Script-Driven Imagery

We utilized procedures for constructing narrative scripts of fear-relevant situations for imagery inductions (Lang, 1979; Lang et al., 1983). The experimenter asked the participant to complete an imagery construction form, which entailed identifying a situation that would be one of the participant’s greatest fears in a dental procedure. The participant was also asked to write down what he/she could imagine seeing, touching, smelling, and hearing in that situation. The experimenter then showed the participant a copy of the PANAS, and asked him/her to identify the five adjectives (from the 20 listed on the PANAS) that he/she would feel in the situation, and then to place the adjectives in order from 1 to 5 (1 = *most prominent feeling*, 5 = *least prominent feeling*). Next, the experimenter showed the participant the 20 physical symptoms of a panic attack that appear in the ADIS interview and asked the participant to identify the five panic symptoms that he/she would feel in the situation, and then to place the symptoms in order from 1 to 5 (1 = *most prominent feeling*, 5 = *least prominent feeling*). The experimenter used the participant’s ideographically identified situation (and accompanying sensory details), accompanying affective experiences, and panic symptoms to construct a narrative imagery script according to a standardized template provided by Lang et al. (1983), as follows:

You are (*Situation*). You notice that you are feeling (*PANAS 5*) and (*PANAS 4*). As you look around, you see (*Sight Item*). You smell (*Smell Item*). You hear (*Hearing Item*). As you are noticing all of the sights, smells, and sounds you are experiencing, you notice that you are also feeling (*PANAS 3*), (*PANAS 2*), and (*PANAS 1*). You notice that (*Panic 5*), (*Panic 4*), and (*Panic 3*). You also notice that you (*Panic 2*) and (*Panic 1*). For the next several minutes, please continue to imagine, as intensely and as vividly as possible, that you are (*Situation*).

For the imaginal exposure exercise, the experimenter asked the participant to close his/her eyes and listen to the experimenter read the idiographic narrative script while imagining the situation, emotions, and sensations as vividly and intensely as possible. Participants were asked to provide 0–100 distress (SUDS) ratings at five time points: just prior to the reading, immediately after the reading, and after 1, 2, and 3 min of continued imagery. Imaginal simulations of anxiety-provoking situations have been shown to elicit subjective and physiological responses (e.g., McDonagh-Coyle et al., 2001), which decrease following exposure therapy (Wangelin and Tuerk, 2015). Such simulations of dental anxiety-relevant cues have also been shown to decrease anxiety prior to and following dental visits (Armitage and Reidy, 2012).

### Procedure

The study was conducted in compliance with the Institutional Review Boards at both sites. Upon arriving at the laboratory, participants provided written informed consent and then completed the clinician interviews. Next, the experimenter guided participants through the completion of forms used to create and tailor a narrative that was relevant to their idiographic dental fears for the script-driven imagery. Participants then completed a battery of self-report measures while the experimenter constructed the imagery scenes based on the situational, physiological, cognitive, and affective stimuli provided by each participant. Following completion of the questionnaires, participants completed the three computerized attention tasks, after which the experimenter guided them through the imaginal exposure task in which they vividly imagined personally feared dental situations as per the scripts constructed and read by the experimenter.

## Results

### Data Reduction

Reaction time data were included in analyses for trials on which responses were (a) accurate, (b) within 2 SD of that participant’s mean response latency (Reese et al., 2010), and (c) between 50 and 1,200 ms (Amir et al., 2003). In addition, participant-level mean reaction time data that were determined to be outliers relative to the overall means for that type of trial across the sample were converted in accordance with the Windsor method to one unit (10 ms) above the next most extreme value. This resulted in Windsorized data from two participants on a total of four trial types (e.g., validly cued dental pictures at pre-training). Mean response latencies at pre-training and post-training are provided in Table 2.

**TABLE 2:** Response Latencies [M (SD) in Milliseconds].

	**Attention training**	**Control**	**Entire sample**
Pre-training			
Dental images			
Validly cued	415.94 (74.06)	388.73 (51.56)	402.86 (65.08)
Invalidly cued	449.37 (79.69)	440.41 (65.28)	445.06 (72.55)
Neutral images			
Validly cued	421.66 (82.38)	399.07 (53.57)	410.80 (70.29)
Invalidly cued	448.76 (79.47)	438.59 (61.87)	443.87 (71.05)
Post-training			
Dental images			
Validly cued	397.56 (84.91)	369.33 (63.79)	383.17 (75.49)
Invalidly cued	445.36 (70.84)	448.06 (72.31)	446.74 (70.89)
Neutral images			
Validly cued	406.21 (86.52)	376.02 (62.99)	390.82 (76.22)
Invalidly cued	446.43 (77.40)	437.48 (65.26)	441.87 (70.89)

*53 participants completed the study. However, due to missing data, *N* = 52 for pre-training reaction time data, and *N* = 51 for post-training reaction time data (*n* = 25 and *n* = 26 in the training and control conditions, respectively).*

### Pre-training

Pre-training mean response latencies were examined using a 2 (Cue: Valid/Invalid) × 2 (Picture: Dental/Neutral) ANOVA with repeated measures on both factors. There was a main effect of Cue such that participants were slower to detect invalidly cued than validly cued probes, *F*(1, 51) = 46.13, *p* < 0.001, η_p_^2^ = 0.48, and CI_90__%_ [0.30,0.59]. There was also a trend indicating that this effect was modified by a Cue × Picture interaction, *F*(1, 51) = 3.70, *p* = 0.06, η_p_^2^ = 0.07, and CI_90__%_ [0,0.20]. Analyses of simple effects indicated that participants were quicker to detect validly cued probes that followed dental pictures than those that followed neutral pictures, *t*(51) = 2.97, *p* = 0.005, *d* = 0.41, and CI_95__%_ [0.13, 0.69], but the reaction time for invalidly cued probes did not differ as a function of picture type, *t*(51) = 0.33, *p* = 0.74, *d* = 0.05, and CI_95__%_ [-0.23, 0.32].

### Post-training

Post-training mean response latencies were examined using a 2 (Group: Training/Control) × 2 (Cue: Valid/Invalid) × 2 (Picture: Dental/Neutral) ANOVA with repeated measures on the last two factors. There was a main effect of Cue such that participants were slower to detect invalidly cued than validly cued probes, *F*(1, 49) = 52.89, *p* < 0.001, η_p_^2^ = 0.52, and CI_90__%_ [0.35,0.63]. In addition, there was still a Cue × Picture interaction indicating attentional bias regardless of condition, and the effect post-training was significant, *F*(1, 49) = 6.99, *p* = 0.01, η_p_^2^ = 0.13, and CI_90__%_ [0.02,0.27]. Again, analyses of simple effects indicated that participants were quicker to detect validly cued probes that followed dental pictures than those that followed neutral pictures, *t*(50) = 3.61, *p* = 0.001, *d* = 0.51, and CI_95__%_ [0.21, 0.80], but the reaction time for invalidly cued probes did not differ as a function of picture type, *t*(50) = -1.10, *p* = 0.28, *d* = -0.15, and CI_95__%_ [-0.43, 0.12]. Hence, training did not eliminate attentional bias. No other effects in the ANOVA were significant.

**FIGURE 1:**
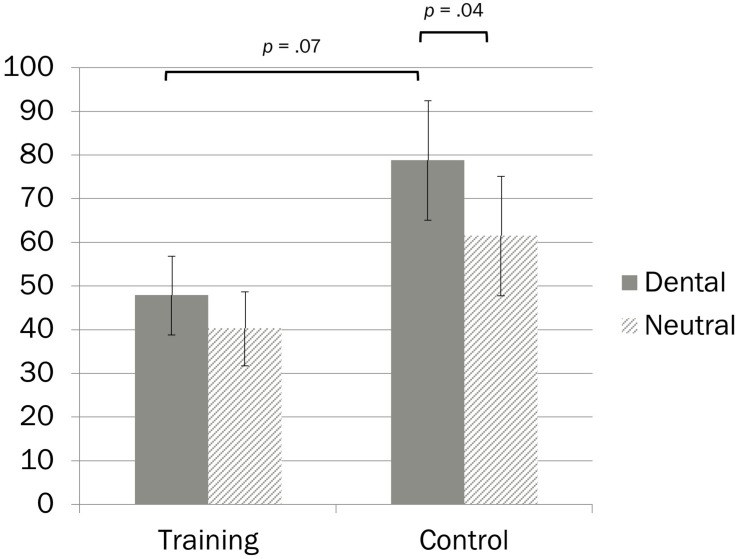
Cue dependency scores (milliseconds) as a function of experimental condition and cue type. Error bars represent 1 *SE*.

Another metric commonly used to examine cue dependency is the *validity effect*, calculated as the difference between response latencies for invalidly cued trials and validly cued trials (Compton, 2000). The validity effect indicates the degree to which participants are influenced by cue location. Rather than simply comparing response latencies separately for validly and invalidly cued trials (as in the simple effects analyses), comparing groups on the validity effect tests potential differences between *slowness on invalidly cued trials* relative to *quickness on validly cued trials*. Validity effect data are presented in Figure 1. Within-group differences in validity effect scores as a function of picture type were examined via paired samples *t*-tests, and group differences in validity effect scores were examined via independent samples *t*-tests. In the attention training group, validity effect scores did not differ as a function of picture type, indicating that the influence of cue location on RT did not depend on picture content, *t*(24) = 1.60, *p* = 0.12, *d* = 0.32, and CI_95__%_ [-0.09, 0.72]. In contrast, the control group had higher validity effect scores (i.e., was more influenced by cue location) for dental than neutral pictures, *t*(25) = 2.16, *p* = 0.04, *d* = 0.42, and CI_95__%_ [0.02, 0.82]. Similarly, group comparisons revealed a trend indicating that the attention training group had lower validity effect scores (i.e., was less influenced by cue location) than did the control group for dental pictures, *t*(49) = 1.87, *p* = 0.067, *d* = 0.52, and CI_95__%_ [-0.04, 1.08], but not for neutral pictures, *t*(49) = 1.31, *p* = 0.197, *d* = 0.37, and CI_95__%_ [-0.19, 0.92].

### Imagery Task

Training condition did not predict distress during the imagery task. First, the training groups did not differ on SUDS ratings immediately prior to commencing the imagery task, *t*(51) = 0.91, *p* = 0.37, *d* = 0.25, and CI_95__%_ [-0.29, 0.79]. Second, post-imagery SUDS ratings were examined via a 2 (Group: Training/Control) × 4 (post-task SUDS ratings time points) ANOVA with repeated measures on the last factor, covarying pre-task SUDS. Mauchly’s test of sphericity was significant, *p* < 0.001, so the Huynh–Feldt correction was applied. There were no significant main effects of Group [*F*(1, 50) = 0.01, *p* = 0.93, and η_p_^2^ < 0.001], or time [*F*(2.34, 117.03) = 0.37, *p* = 0.72, and η_p_^2^ = 0.007], nor was the interaction significant [*F*(2.34, 117.03) = 0.11, *p* = 0.92, and η_p_^2^ = 0.002]. The average peak SUDS rating was 76.64 (SD = 21.61) across the sample, and similar within both groups [for the training group, 77.78 (SD = 19.34); for the control group, 75.46 (SD = 24.08)]. This indicates that the imagery task induced considerable anxiety and therefore the reason training did not impact anxiety was not because participants were insufficiently anxious.

Across the entire sample, validity effect scores for dental pictures were correlated with distress immediately before commencing the imagery task (*r* = 0.47, *p* = 0.001), but not at any point after the imagery task (*r’*s < 0.20, *p’*s > 0.17). However, the pattern was similar for validity effect scores for neutral pictures, which likewise predicted distress immediately before commencing the imagery task (*r* = 0.41, *p* = 0.003), but not at any point thereafter (|*r*| ‘*s* < 0.09, *p’*s > 0.56).

## Discussion

The aims of this investigation were to examine whether individuals with dental anxiety have threat-relevant biased attention and to test the impact of an experimental one-session attention training intervention on attentional bias and subjective anxiety during an imagery task. Several previous studies demonstrate an attentional bias in individuals with dental anxiety (Muris et al., 1995; Ries, 1997; Johnsen et al., 2003); however, those studies used the Stroop paradigm, which confounds the potential roles of attentional bias and NA in influencing reaction times. In the present study, there was consistent evidence both before and after the training manipulation of attentional bias toward threat as measured using the Posner paradigm.

Previously, researchers used the Posner task to differentiate between facilitated attention toward, and difficulty disengaging attention from, threat (e.g., Cisler and Koster, 2010). Specifically, facilitated attention toward threat was seen as evident when participants detected validly cued probes that follow threat cues more quickly than those that follow neutral cues. In contrast, difficulty disengaging from threat was inferred when participants detected invalidly cued probes that follow threat cues more slowly than those that follow neutral cues. On the basis of this reasoning, the present study would seem to indicate the presence of facilitated attention toward threat, but not difficulty disengaging from threat, in dental anxiety. More recently, however, researchers have identified limitations in the ability of this paradigm to test the attentional bias mechanism, and demonstrated that alternative processes may even reverse the results (e.g., Mogg et al., 2008; Grafton and MacLeod, 2014; Mogg and Bradley, 2016). Therefore, the present results indicate attentional bias, but cannot elucidate the nature of the mechanism.

The results of the attention training manipulation were mixed. Contrary to expectations, training did not eliminate attentional bias, and the patterns of cue dependency (i.e., validity effect) scores for dental versus neutral trials appeared similar in the two groups. However, cue dependency scores for participants in the attention training group did not depend on picture type (dental versus neutral), but cue dependency scores for those in the control group were larger for dental than neutral picture cues. Furthermore, training reduced overall cue dependency, which may indicate that it was successful in training participants to allocate their attention more effectively in general, whether or not it specifically reduced threat-relevant bias. Although there was no impact of training on anxiety after the imagery task, the ability to allocate attention without being as affected by the cue location—for both threat and neutral cues—predicted less anticipatory anxiety before the task.

Taken together, it is possible that the effect of training was to enhance attentional control, not to reduce bias. If participants were more able to focus their attention on the fixation point, this would result not only in smaller cue dependency scores for both dental and neutral pictures, but also in smaller differences between cue dependency scores for dental versus neutral pictures. At the least, the impact of attention on anxiety prior to the imagery task must result from overall attentional control rather than something specific to threat content because cue dependency scores for both dental and neutral pictures predicted pre-imagery distress. If so, these results are consistent with other studies that demonstrate that attention modification procedures have similar effects whether participants are trained to direct their attention away from, or toward, threat (e.g., Klumpp and Amir, 2010), implying that the critical element is overall attentional control. In fact, attentional control has been associated with improvements in anxiety outcomes even in investigations that did not find evidence for the superiority of attention training relative to control conditions (for a review, see Mogg and Bradley, 2016).

Attention training did not impact subjective anxiety before or following an imagery task. Importantly, Najmi and Amir (2010) demonstrated that attentional bias modification can influence behavioral outcomes without reducing subjective distress. Specifically, they implemented an attention training protocol similar to the one used in the present study with individuals who had subclinical symptoms of OCD focused on contamination. They found that although attention training had no impact on anxiety or OCD symptoms, participants who received training completed more steps on a contamination behavioral approach task. Therefore, it is possible that individuals with dental anxiety may be more willing to visit the dentist or complete dental procedures following attention training, even if such events do not lead to less anxiety. Although the availability of such a brief and portable intervention capable of promoting healthy behavioral changes despite maintained anxiety would offer a considerable public health benefit, the efficacy of such an intervention for dental anxiety awaits empirical investigation. In addition, testing the impact of attention training before an actual dental procedure could raise the emotional salience of the threat cues and generally raise the degree of environmental threat, which may facilitate threat detection (e.g., Cornwell et al., 2011), and impair cognitive flexibility related to attention (e.g., Cornwell et al., 2012).

One previous investigation examined attention training in a dental clinic, where participants completed attention training, control, or distraction tasks while awaiting an appointment (Horovitz et al., 2016). The authors found that the attention training condition was associated with maintained anxiety before and after dental treatment, whereas the control and distraction conditions were associated with decreased anxiety before and after dental treatment, respectively. Of note, the researchers specifically excluded participants with high levels of dental anxiety, so these results may not be relevant to individuals with clinically significant levels of dental anxiety, for whom an intervention is more necessary and relevant. Moreover, cognitive bias modification procedures may produce larger effects in clinical samples (e.g., Hallion and Ruscio, 2011). Therefore, an important next step is to test the efficacy of attention training with highly dentally anxious participants in a dental setting, using a behavioral approach outcome assessment.

There were several limitations to the present study. First, due to an administrative error, formal diagnoses of dental phobia were not available. However, the clinician ratings of interference and distress were consistent with the likelihood that nearly all participants met criteria for current dental phobia. Second, although all participants exceeded the clinical cutoff score on a measure of dental anxiety, most participants were referred from university dental clinics. Presumably, individuals with the most severe dental anxiety avoid going to the dentist altogether, in which case the most severe patients would not have had the opportunity to participate. It is important to include even these most severe individuals in future investigations, particularly because such individuals might benefit the most, and/or might improve even in the absence of exposure-based interventions. Third, in spite of random assignment, the experimental groups differed in racial composition. As described, it is not appropriate to covary a demographic variable in a randomized trial especially without a theoretical or empirical reason to expect it to influence outcomes of interest. Nevertheless, the group difference in racial composition presents a conceivable alternative hypothesis or confound. Fourth, participants were not seated at a standardized distance from the computers, and therefore we cannot report visual angle. Fifth, without a comparison group of individuals low in dental anxiety, one cannot be certain that the attentional bias toward dental cues relative to neutral cues is specific to individuals high in dental anxiety.

In conclusion, this is the first study to demonstrate that high dental anxiety is associated with threat-relevant attentional bias using the Posner paradigm. The results of attention training were mixed, but overall seem to indicate that training led to enhanced attentional control; it is less clear whether it led to decreased attentional bias toward threat. Attention training did not reduce anxiety during an imagery task, although overall attentional control was associated with less anticipatory anxiety before the task. Considering that the literature is inconclusive about whether the remedial effects of attention training require that it reduces attentional bias (cf. MacLeod and Clarke, 2015; Mogg and Bradley, 2016), and that attention training can increase the willingness to approach feared situations without reducing anxiety (Najmi and Amir, 2010), future research should examine the effects of attention training on behavioral outcomes such as completing a feared dental procedure.

## Data Availability Statement

The datasets generated for this study are available on request to the corresponding author.

## Ethics Statement

The studies involving human participants were reviewed and approved by Institutional Review Boards at Nova Southeastern University and the University of Illinois at Chicago. The participants provided their written informed consent to participate in this study.

## Author Contributions

JS and EB designed the study, attained funding for it, supervised data collection at the two sites, analyzed and interpreted the data, and prepared the manuscript. MF collected the data and contributed to the manuscript.

## Conflict of Interest

The authors declare that the research was conducted in the absence of any commercial or financial relationships that could be construed as a potential conflict of interest.

## Footnotes

1 de Jongh et al. (1995b) found that trait dental anxiety was unrelated to the length of time participants directed their gaze at dental threat-related pictures relative to neutral pictures. However, the participants were unselected vis-à-vis dental anxiety and the task consisted of prolonged exposure to pictures, rather than paradigms that detect attention allocation at early stages of information processing.

2 Despite random assignment, the experimental groups differed in racial composition. However, there is no theory or evidence linking race to the outcomes of interest, and race was not associated with any measure of response latency pre-training or post-training. It is also conceptually problematic to covary a demographic variable that differs between groups because doing so removes variance associated with the experimental manipulation itself, thereby distorting the independent variable of interest (viz. experimental condition; Miller and Chapman, 2001). More generally, others have argued that in randomized trials one should not covary baseline differences on the basis of whether they are significant (Moher et al., 2010), and even that it is inappropriate to conduct significance tests of such variables following randomization altogether (e.g., Begg, 1990; Pocock et al., 2002; Moher et al., 2010). We therefore did not covary race in any analyses.
